# Compound heterozygous variants in *CFTR* with potentially reducing ATP‐binding ability identified in Chinese infertile brothers with isolated congenital bilateral absence of vas deferens

**DOI:** 10.1002/mgg3.2249

**Published:** 2023-07-24

**Authors:** Shi Shengjia, Wang Lei, Wang Tianwei, Wang Hongmei, Shi Juanzi, Qiao Sen

**Affiliations:** ^1^ Reproductive Center Northwest Women's and Children's Hospital Xi'an China; ^2^ School of Medicine Southeast University Nanjing China

**Keywords:** azoospermia, *CFTR* mutation, compound heterozygous variant, congenital bilateral absence of vas deferens, male infertility

## Abstract

**Background:**

Isolated congenital bilateral absence of vas deferens (iCBAVD) in men results in obstructive azoospermia and is mainly caused by pathogenic variants in cystic fibrosis transmembrane conductance regulator (*CFTR*) or adhesion G protein‐coupled receptor G2 (*ADGRG2*)*.*

**Methods:**

The next‐generation sequencing (NGS) was used to screen the mutations in the proband, and Sanger sequencings were performed to validate the compound heterozygous variant of *CFTR* in his family members*.* Protein structure simulation was performed to discover the potential pathological mechanism.

**Results:**

This study reported novel compound heterozygous *CFTR* mutations (NM:000492.4, Intron: 5T; c.3965_3969dupTTGGG: p.R1325Gfs*5) in two brothers with obstructive azoospermia. The compound heterozygous *CFTR* mutations were first screened out by NGS in an infertile male patient who exhibited iCBAVD from a nonconsanguineous Chinese family. Histological analysis of the testicular biopsy from this patient revealed normal spermatogenesis and mature spermatozoa were observed in the seminiferous tubules. Surprisingly, the same compound heterozygous *CFTR* mutations were also observed in his brothers who also exhibited iCBAVD, with their parents being a heterozygous carrier for the mutations, as verified by Sanger sequencing. Protein structure simulation revealed that these mutations potentially led to impaired ATP‐binding ability of CFTR.

**Conclusion:**

We identified novel compound heterozygous *CFTR* mutations in two brothers and summarized the literature regarding *CFTR* mutation and male infertility. Our study may contribute to the genetic diagnosis of iCBAVD and future genetic counseling.

## Graphical abstract

This study reported novel compound heterozygous CFTR mutations (NM:000492.4, Intron: 5T; c.3965_3969dupTTGGG: p.R1325Gfs*5) in two brothers with obstructive azoospermia. Protein structure simulation revealed that these mutations potentially led to impaired ATP‐binding ability of CFTR.

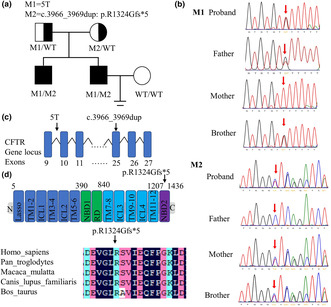

## 1 INTRODUCTION

Male infertility is defined as the inability to make their partner achieve pregnancy after 12 months of regular unprotected sexual intercourse, and about 10%–15% of male infertility patients is characterized as the absence of sperm in semen (Caroppo & Colpi, 2021; Saberiyan et al., 2022). Approximately, up to 25% of obstructive azoospermia is caused by congenital bilateral or unilateral absence of vas deferens (Cheng et al., 2022; Wang et al., 2020).

Cystic fibrosis (CF) is an autosomal recessive inherited disorder, which involves mutations of the cystic fibrosis transmembrane conductance regulator (*CFTR*, OMIM 602,421) gene on chromosome 7 (Cioppi et al., 2021; Fedder et al., 2021; Gaikwad et al., 2020). CFTR is a member of ATP‐binding cassette (ABC) superfamily. Unlike other family members which actively pump substrates against their chemical gradients by ATP hydrolysis, CFTR serves as ATP‐gated ion channel (Gadsby et al., 2006). Like a typical ABC transporter, CFTR consists of two transmembrane domains (TMDs) that form the translocation pathway and two cytoplasmic nucleotide‐binding domains (NBDs; Gadsby et al., 2006). Binding of ATP to both NBD is important for pore opening and therefore is critical for CFTR to exert its function.

Interestingly, though, CF was relatively rare in the Chinese population, the *CFTR* gene variants were not as rare as once believed in obstructive azoospermia based on congenital bilateral or unilateral absence of vas deferens in the Chinese population (Cheng et al., 2022; Wang et al., 2020). Most of obstructive azoospermia patients who carry CBAVD and *CFTR* gene variants exhibit no symptoms related to CF in China, which is defined as isolated CBAVD (iCBAVD; Cai & Li, 2022). Interestingly, the mutation spectrums of Caucasian or Chinese CBAVD patients are diversified. Unlike Caucasian CBAVD patients which approximately 70% cases carried p.F508del *CFTR* mutation (Fiore et al., 2020; Sanseverino et al., 2022), c.2909G>A variant was the most common *CFTR* mutation type in Chinese CBAVD patients, but only accounting for 11% of cases (Cai & Li, 2022; Shao et al., 2020). Furthermore, though the mutation types of *CFTR* in Caucasian CBAVD patients have been well established, Chinese *CFTR* mutation profiles of iCBAVD patients are still required to be further characterized.

In the case reported herein, we reported two brothers with iCBAVD resulted in obstructive azoospermia. Both of the brothers harbored novel compound heterozygous mutations in *CFTR* (5T, inherited from the father and c.3965_3969dupTTGGG, p.R1325Gfs*5 inherited from the mother). As c.3965_3969dupTTGGG is not currently listed in the Cystic Fibrosis Mutation Database, this information, regarding the iCBAVD‐causing mutations in two Chinese patients, is of interest.

## 2 METHODS

### 2.1 Ethical compliance

The study was approved by the Ethics Review Board of the Northwest Women's and Children's Hospital (ethical review number: 2022020). Written informed consent was obtained from the participants for the use of their specimens and anonymized data for research purpose.

### 2.2 Patients and medical exome sequencing

The proband was a 30‐year‐old man from a non‐consanguineous Han Chinese family. He was recruited to identify genetic risk factors at Reproductive center of Northwest Women's and Children's Hospital due to the absence of sperm in semen. The routine clinical examination exceeded the usual causes for azoospermia including hormone levels, chromosomal aberration, and Y chromosome microdeletion. However, physical examination, seminal plasma biochemistry analysis, and transrectal ultrasound found bilateral absence of vas deferens in the proband, which is clinically considered as a risk factor for azoospermia. Based on these findings, medical exome sequencing (Amcarelab, Guangzhou, China) was performed on the proband and his wife to identify genetic causes and assess the risk of genetic abnormalities in offspring. Sanger sequencing was further used to validate pathogenic variants in his family. For the structural simulation, human *CFTR* (NCBI Reference Sequence: NC_000007.14) cDNA was used as template. Simulated protein structure was presented by using PyMOL Molecular Graphics System (version 1.5.0.4, Schrodinger, New York, NY, USA) by Coot30 (Emsley et al., 2010), based on the coordinates of human *CFTR* (Liu et al., 2017; PDB: 5UAK).

## 3 RESULTS

### 3.1 Case presentation

The proband was a 30‐year‐old and referred the hospital for having been infertile for 1 year after marriage. Semen examination showed a total absence of sperm with decreased pH value (6.0) and a volume (1.7 mL) closed to normal threshold. Physical examination and scrotal ultrasound showed bilateral absence of vas deferens and normal epididymis structure in scrotum. The patients had normal karyotype and no microdeletions in the Y chromosome. Seminal plasma biochemistry analysis indicated that seminal fructose level (1.36 μmol a single ejaculate) and neutral alpha‐glucosidase level (14.62 mU a single ejaculate) were reduced. Transrectal ultrasound also found bilateral absence of vas deferens in pelvic cavity and volumes of bilateral seminal vesicles were also decreased. The serum follicle‐stimulating hormone (FSH, 3.51 IU/L), luteinizing hormone (LH, 2.28 IU/L), and total testosterone (T, 314.85 ng/dL) indicate the proband might have normal spermatogenic function. Consistently, the testicular sperm aspiration (TESA) showed motile sperms in his right testicle. More importantly, this proband showed no symptoms with regarding respiratory tract and gastrointestinal systems related to CF. Therefore, this proband diagnosed with iCBAVD, and the proband's wife who did not carry the pathogenic variation of *CFTR* received TESA and intracytoplasmic sperm injection (ICSI) treatment for assisted reproduction procedures. On the day of ovum picking up, 16 oocytes were retrieved from his wife, and 10 of 16 oocytes were successfully fertilization after ICSI treatment. Eventually, six cleavage embryos were formed on the 3‐day postfertilization, and two blastocytes were formed from five cleavage embryos on the 5‐day postfertilization after freezing one high‐quality cleavage embryo. After transferred one blastocyte, the proband's wife was successfully pregnant.

### 3.2 Family description

**FIGURE 1:**
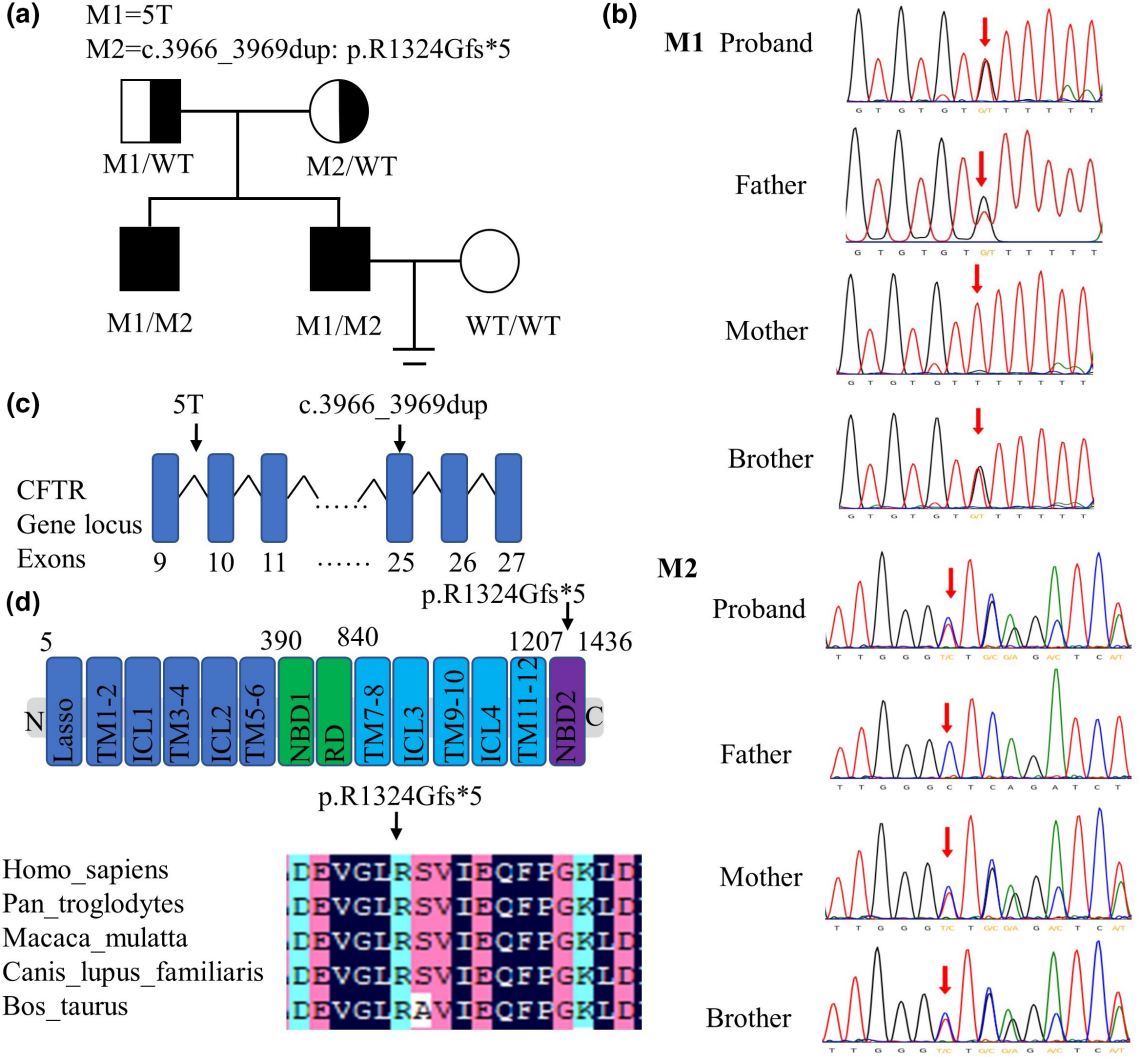
Pedigree and mutation analysis of family with CBVAD. (a) Pedigree of family with CBVAD. WT, wild type CFTR gene; M1: CFTR gene with 5T mutation; M2 CFTR gene with c.3966_3969 dup mutation. (b) Sanger sequencing results of 5T and c.3966_3969 dup for the family. (c) Location of mutations in CFTR gene. (d) Location and conservation of p. R1324Gfs*5 in CFTR protein. CFTR NCBI Reference Sequence: NC_000007.14.

The two brothers exhibited the same symptoms of azoospermia and iCBAVD, whereas their father's sperm count and vas deferens in scrotum was not found abnormal by semen analysis and physical examination. Meanwhile, their mother showed no symptoms of CF or genital system defects (Figure 1a).

### 3.3 Identification of novel compound heterozygous mutations in *CFTR*

Through manual interpretation of the medical exome sequencing results and excluding the benign, likely benign, and uncertain significance *CFTR* mutations, we identified a novel compound heterozygous *CFTR* mutations which consisted of the 5T mutation in intron 9 (HG19: chr7:117188685–117188689) and c.3965_3969dupTTGGG in exon 25 (HG19: chr7:117304741) which was a novel mutation has not yet been recorded in the *CFTR* mutation database (http://www.genet.stickkids.on.ca/cftr/app). Sanger sequencing of these *CFTR* mutations was then performed, which further confirmed infertility phenotype co‐segregated with *CFTR* compound heterozygous mutations in the pedigree. His father was a heterozygous carrier of the *CFTR* 5T variant, his mother carried another variant in the *CFTR* c.3965_3969dupTTGGG, and his brother carried the same compound heterozygous *CFTR* mutations as the proband (Figure 1b).

The 5T variant gives rise to 11TG‐7T being replaced by 12TG‐5T, which resulted in gene splicing failure in intron 9 of *CFTR* gene (Figure 1c, Figure S1). The c.3965_3969dupTTGGG variants cause frameshift mutation with alteration of arginine to glycine from 1325 to 1330 and introduced a new terminating TAA at the position of 1326 (Figure 1c,d, Figure S2). According to the ACMG guidelines, both 5T and c.3965_3969dupTTGGG variants are classified as “likely pathogenic (LP)” (5T variant: PS3+PM2+PM3; c.3965_3969dupTTGGG: PM2+PM3+PP1+PP3‐+PVS1). All the above results indicated that the compound heterozygous variants of *CFTR* were very likely to be the genetic causes of the two brothers' CBAVD phenotypes.

### 3.4 Both mutations render CFTR proteins low ATP‐binding ability

**FIGURE 2:**
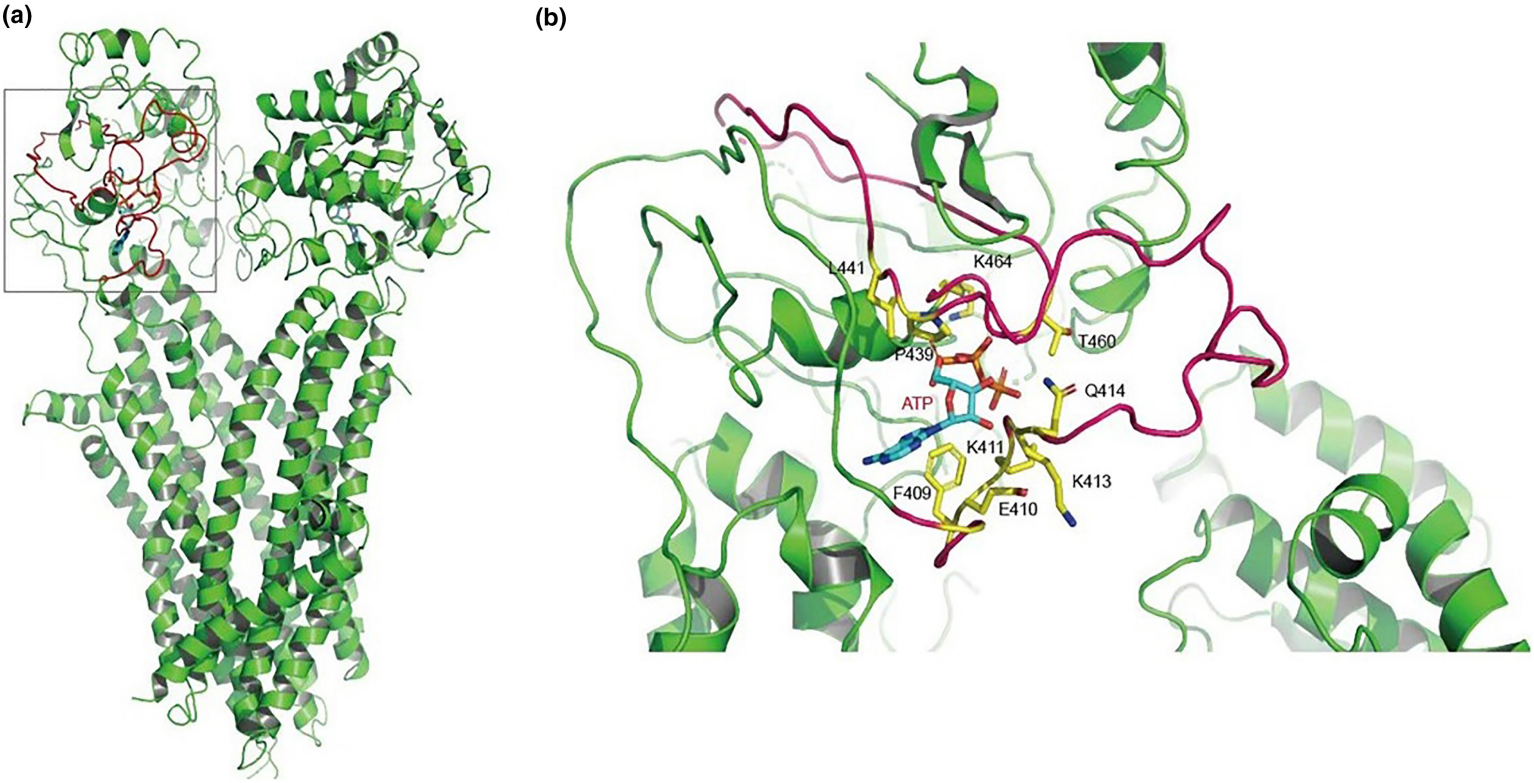
Disease association, structure representation, and sequence alignment of CFTR variants: the loss of exon 10. (a) Human CFTR protein structure (PDB: 5UAK). (b) Structural representation of interactions between CFTR and ATP. The figure was prepared with Pymol using the coordinates from PDB. ATP, exon 10, and interacting amino acids are depicted in blue, red, and yellow, respectively.

**FIGURE 3:**
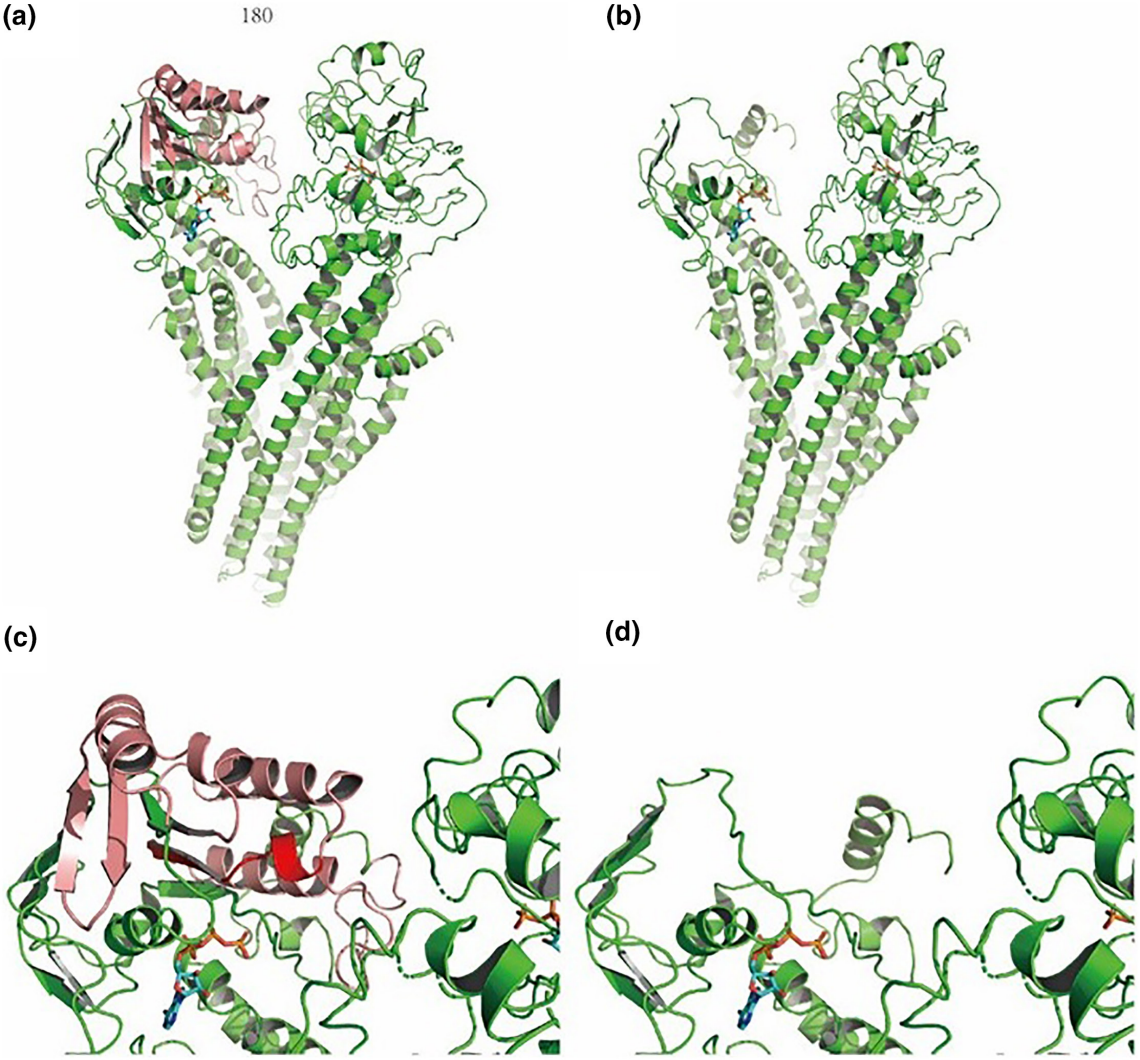
Disease association, structure representation, and sequence alignment of CFTR variants: the loss of C terminal. (a) The overall structure of human CFTR (PDB: 5UAK); (b) the overall structure of human CFTR with the loss of C terminal (PDB: 5UAK); (c) the interaction between ATP (blue) and C terminal of CFTR (in red); (d) the loss of C terminal caused the other binding site with ATP of CFTR.

Previous reports on the protein structure of human CFTR revealed the architecture of the ion pore, as well as the structural basis for CFTR's channel activity. The proposed structure of CFTR provides a suitable tool for modeling structural consequences of genomic mutations discovered in human patients (Figure 2a). We performed simulations on this published CFTR structure based on the 5T mutation or c.3965_3969dupTTGGG mutation. The amino acids at F409, E410, K411, Q414, P439, L441, T460, and K464 positions in human CFTR had interactions with ATP. Especially, the cyclic side chains forming strong hydrophobic interactions. The loss of exon 10 caused the missing of these interactions, therefore led to loosen binding or even lose the binding with ATP, eventually impaired the CFTF function (Figure 2b). c.3965_3969dupTTGGG mutation caused frameshift and then premature termination of translation in C terminal (Figures S3 and S4). The missing of C terminal impaired the other binding site with ATP of CFTR (Figure 3). In total, this novel compound heterozygous mutations in CFTR impaired both the binding sites with ATP.

## 4 DISCUSSION

In the current study, we identified a rare frameshift mutation (NM_000492.4: c.3965‐3969dupTTGGG; p.R1325Gfs*5) leading to failure in the production of a full‐length CFTR protein of which only cytosolic nucleotide‐binding domain 2 (NBD2) of C‐terminated truncated (Hwang et al., 2018). As known to us all, CFTR is a member of ABC transporter superfamily and has a peptide chain of 1480 amino acids translated from 27 exons, forming a phosphorylated chloride channel (Bareil & Bergougnoux, 2020). More specifically, CFTR protein consisted of two transmembrane domains (TMDs) which form the channel pore, two cytosolic NBDs which drive channel gating, and unstructured regulatory domain between NBD1 and TMD2 which control channel activity via PKA‐mediated phosphorylation (Csanady et al., 2019). Interestingly, the mutation in NBD1, such as p.F508del mutation, was proved to lead to energetic and kinetic instability of CFTR protein and CBAVD (Smirnikhina et al., 2020). But little is known concerning the relationship between mutation in NBD2 and the phenotype of iCBAVD.

Here, we show the frameshift mutation in NBD2 of CFTR which is highly conserved among different species (PP3) also results in iCBAVD. The population variation rate of this frameshift in NBD2 of CFTR causing a null variant (PVS1) is 0 in public databases (PM2). Co‐segregation analysis in the family with two affected patients (PP1) confirmed that both patients harbored the frameshift mutation in *trans* with 12TG‐5T allele (PM3), which may cause dysfunctional CFTR protein without channel activity and resulted in iCBAVD. Thus, this rare frameshift mutation of CFTR should be classified as a likely pathogenic variant according to ACMG guidelines (Richards et al., 2015).

It had been reported 5T allele in intron 9 of *CFTR* gene was one of the most common pathologic variants in Chinese CBAVD population (Huang et al., 2008; Zhao et al., 2022), which is different from the Caucasian CBAVD population. Previous study had proved the poly‐T polymorphism in intron 9 of *CFTR* may alter the splicing pattern of exon 10 and result in reduction of CFTR protein production (Chu et al., 1993). There are three alleles at the poly‐T locus, 5T (5%), 7T (84%), and 9T (11%). Among the three forms of poly‐T, homozygous 5T is the main cause of skipping of exon 10, but homozygous 7T and 9T are less likely to result in skipping of exon 10 of *CFTR* (Tabaripour et al., 2012). Notably, exon 10 encodes 60 amino acids of NBD1, and CFTR protein without these amino acids would lose the function of chloride channel (Hojati et al., 2012). Thus, it further reduces the permeability of chloride ion in the ductal epithelial cell membrane and increases the reabsorption of sodium ion, which results in an increase in the concentration of sodium and chloride ion in extracellular secretion and eventually leads to CF or iCBAVD phenotypes. Interestingly, the pathogenicity 5T variants are characterized by incomplete exodominance, which means the compound heterozygous variants of 5T and a severe *CFTR* mutation *in trans* could still be detected in fertile healthy population (Claustres, 2005). Cuppens found the TG repeats which is adjacent to poly‐T could also reduce the splicing efficiency of exon 10, and the more TG repeats, the higher penetrance of poly‐T (Cuppens et al., 1998). Compared with fertile healthy population, 13TG‐5T and 12TG‐5T are more common in CBAVD patients.

Interestingly, CBAVD could occur in an isolated form or in company with other typical manifestations of CF. Though *CFTR* gene is the main genetic cause of CBAVD, different biallelic combinations of *CFTR* variants may lead to different clinical manifestations. Previous studies indicated only men with homozygous of two severe pathogenic *CFTR* mutations could develop the disease CF, men with *CFTR* compound heterozygote or a single pathogenic *CFTR* exon mutation, in some cases combined with a 5T splice variant, may only have iCBAVD phenotype without symptoms from the respiratory tract and gastrointestinal system (Yu et al., 2012). More importantly, the 5T mutation is commonly reported with high frequency (44.4%) in Chinese CBAVD patients (Wu et al., 2004). A previous literature reported a proband and his brother carried heterozygote composite *CFTR* variants, including 5T and c.50dupT. The proband was identified as CUAVD, and his brother was identified as CBAVD. Both of the patients showed no symptoms of CF and have bilateral normal kidneys (Ge et al., 2019). Thus, we speculate the Chinese men carrying a compound heterozygote which consisted by 5T and other pathogenic *CFTR* variant most likely do not present a CF phenotype and only present as iCBAVD. This hypothesis needs further validation in a large iCBAVD patient cohort in China.

In conclusion, we identified a rare frameshift variant in trans with IVS9‐12TG‐5T allele of *CFTR* in a Chinese pedigree with two iCBAVD patients. Our study has broadened the *CFTR* mutation spectrum in Chinese iCBAVD patients and provided more familial evidence of the pathogenicity of compound heterozygous variants of *CFTR*.

## AUTHOR CONTRIBUTIONS

Shi Shengjia, Wang Lei, Wang Tianwei, and Shi Juanzi recruited subjects and sorted clinical information. Wang Hongmei performed protein structural simulation. Qiao Sen designed the study and wrote the manuscript.

## FUNDING INFORMATION

This work was supported by Xi'an Science and Technology Planning Project (2021JH‐04‐0130) and Health Research Fund Shaanxi Provincial Health Commission (2021E024).

## CONFLICT OF INTEREST STATEMENT

The authors declare that the research was conducted in the absence of any commercial or financial relationships that could be construed as a potential conflict of interest.

## ETHICS STATEMENT

The study was approved by the Ethics Review Board of the Northwest Women's and Children's Hospital (ethical review number: 2022020).

## CONSENT TO PARTICIPATE

Written informed consent was obtained from the participants for the use of their specimens and anonymized data for research purpose.

## CONSENT FOR PUBLICATION

Publication consent was obtained from all authors.

## Supporting information


Figure S1

Figure S2

Figure S3

Figure S4

## Data Availability

The NGS datasets supporting the current study have not been deposited in a public repository because of privacy and ethical restrictions but are available from the corresponding authors on request. All the other original data are present in the published article, and further inquiries can be directed to the corresponding authors.

## References

[mgg32249-bib-0001] Bareil, C. , & Bergougnoux, A. (2020). CFTR gene variants, epidemiology and molecular pathology. Archives de Pédiatrie, 27(Suppl. 1), eS8–eS12. 10.1016/S0929-693X(20)30044-0 32172939

[mgg32249-bib-0002] Cai, Z. , & Li, H. (2022). Congenital bilateral absence of the vas deferens. Frontiers in Genetics, 13, 775123. 10.3389/fgene.2022.775123 35222530 PMC8873976

[mgg32249-bib-0003] Caroppo, E. , & Colpi, G. M. (2021). Prediction models for successful sperm retrieval in patients with non‐obstructive azoospermia undergoing microdissection testicular sperm extraction: Is there any room for further studies? Journal of Clinical Medicine, 10(23), 5538. 10.3390/jcm10235538 34884245 PMC8658396

[mgg32249-bib-0004] Cheng, H. , Yang, S. , Meng, Q. , Zheng, B. , Gu, Y. , Wang, L. , Song, T. , Xu, C. , Wang, G. , Han, M. , Shen, L. , Ding, J. , Li, H. , & Ouyang, J. (2022). Genetic analysis and intracytoplasmic sperm injection outcomes of Chinese patients with congenital bilateral absence of vas deferens. Journal of Assisted Reproduction and Genetics, 39(3), 719–728. 10.1007/s10815-022-02417-z 35119551 PMC8995229

[mgg32249-bib-0005] Chu, C. S. , Trapnell, B. C. , Curristin, S. , Cutting, G. R. , & Crystal, R. G. (1993). Genetic basis of variable exon 9 skipping in cystic fibrosis transmembrane conductance regulator mRNA. Nature Genetics, 3(2), 151–156. 10.1038/ng0293-151 7684646

[mgg32249-bib-0006] Cioppi, F. , Rosta, V. , & Krausz, C. (2021). Genetics of azoospermia. International Journal of Molecular Sciences, 22(6), 3264. 10.3390/ijms22063264 33806855 PMC8004677

[mgg32249-bib-0007] Claustres, M. (2005). Molecular pathology of the CFTR locus in male infertility. Reproductive Biomedicine Online, 10(1), 14–41. 10.1016/s1472-6483(10)60801-2 15705292

[mgg32249-bib-0008] Csanady, L. , Vergani, P. , & Gadsby, D. C. (2019). Structure, gating, and regulation of the CFTR anion channel. Physiological Reviews, 99(1), 707–738. 10.1152/physrev.00007.2018 30516439

[mgg32249-bib-0009] Cuppens, H. , Lin, W. , Jaspers, M. , Costes, B. , Teng, H. , Vankeerberghen, A. , Jorissen, M. , Droogmans, G. , Reynaert, I. , Goossens, M. , Nilius, B. , & Cassiman, J. J. (1998). Polyvariant mutant cystic fibrosis transmembrane conductance regulator genes. The polymorphic (Tg)m locus explains the partial penetrance of the T5 polymorphism as a disease mutation. The Journal of Clinical Investigation, 101(2), 487–496. 10.1172/JCI639 9435322 PMC508589

[mgg32249-bib-0010] Emsley, P. , Lohkamp, B. , Scott, W. G. , & Cowtan, K. (2010). Features and development of coot. Acta Crystallographica. Section D, Biological Crystallography, 66(Pt 4), 486–501. 10.1107/S0907444910007493 20383002 PMC2852313

[mgg32249-bib-0011] Fedder, J. , Jorgensen, M. W. , & Engvad, B. (2021). Prevalence of CBAVD in azoospermic men carrying pathogenic CFTR mutations—Evaluated in a cohort of 639 non‐vasectomized azoospermic men. Andrology, 9(2), 588–598. 10.1111/andr.12925 33095972 PMC7894542

[mgg32249-bib-0012] Fiore, M. , Picco, C. , & Moran, O. (2020). Correctors modify the bicarbonate permeability of F508del‐CFTR. Scientific Reports, 10(1), 8440. 10.1038/s41598-020-65287-4 32439937 PMC7242338

[mgg32249-bib-0013] Gadsby, D. C. , Vergani, P. , & Csanady, L. (2006). The ABC protein turned chloride channel whose failure causes cystic fibrosis. Nature, 440(7083), 477–483. 10.1038/nature04712 16554808 PMC2720541

[mgg32249-bib-0014] Gaikwad, A. , Khan, S. , Kadam, S. , Shah, R. , Kulkarni, V. , Kumaraswamy, R. , Kadam, K. , Dighe, V. , & Gajbhiye, R. (2020). Cystic fibrosis transmembrane conductance regulator‐related male infertility: Relevance of genetic testing & counselling in Indian population. The Indian Journal of Medical Research, 152(6), 575–583. 10.4103/ijmr.IJMR_906_18 34145097 PMC8224163

[mgg32249-bib-0015] Ge, B. , Zhang, M. , Wang, R. , Wang, D. , Li, T. , Li, H. , & Wang, B. (2019). A rare frameshift variant in trans with the IVS9‐5T allele of CFTR in a Chinese pedigree with congenital aplasia of vas deferens. Journal of Assisted Reproduction and Genetics, 36(12), 2541–2545. 10.1007/s10815-019-01617-4 31709488 PMC6911126

[mgg32249-bib-0016] Hojati, Z. , Heidari, S. , & Motovali‐Bashi, M. (2012). Exon 10 CFTR gene mutation in male infertility. Iranian Journal of Reproductive Medicine, 10(4), 315–320. 25246892 PMC4165948

[mgg32249-bib-0017] Huang, Q. , Ding, W. , & Wei, M. X. (2008). Comparative analysis of common CFTR polymorphisms poly‐T, TG‐repeats and M470V in a healthy Chinese population. World Journal of Gastroenterology, 14(12), 1925–1930. 10.3748/wjg.14.1925 18350634 PMC2699602

[mgg32249-bib-0018] Hwang, T. C. , Yeh, J. T. , Zhang, J. , Yu, Y. C. , Yeh, H. I. , & Destefano, S. (2018). Structural mechanisms of CFTR function and dysfunction. The Journal of General Physiology, 150(4), 539–570. 10.1085/jgp.201711946 29581173 PMC5881446

[mgg32249-bib-0019] Liu, F. , Zhang, Z. , Csanady, L. , Gadsby, D. C. , & Chen, J. (2017). Molecular structure of the human CFTR ion channel. Cell, 169(1), 85–95.e88. 10.1016/j.cell.2017.02.024 28340353

[mgg32249-bib-0020] Richards, S. , Aziz, N. , Bale, S. , Bick, D. , Das, S. , Gastier‐Foster, J. , Grody, W. W. , Hegde, M. , Lyon, E. , Spector, E. , Voelkerding, K. , Rehm, H. L. , & ACMG Laboratory Quality Assurance Committee . (2015). Standards and guidelines for the interpretation of sequence variants: A joint consensus recommendation of the American College of Medical Genetics and Genomics and the Association for Molecular Pathology. Genetics in Medicine, 17(5), 405–424. 10.1038/gim.2015.30 25741868 PMC4544753

[mgg32249-bib-0021] Saberiyan, M. , Karimi, E. , Safi, A. , Movahhed, P. , Dehdehi, L. , Haririan, N. , & Mirfakhraie, R. (2022). Circular RNAs: Novel biomarkers in spermatogenesis defects and male infertility. Reproductive Sciences, 30, 62–71. 10.1007/s43032-022-00885-3 35178677

[mgg32249-bib-0022] Sanseverino, P. B. , Hoffmann, A. , Machado, S. , Farias, M. , Michels, M. S. , Sanseverino, M. T. V. , & Marostica, P. J. C. (2022). High‐risk twin pregnancy: Case report of an adolescent patient with cystic fibrosis and systemic lupus erythematosus. Journal of Medical Case Reports, 16(1), 230. 10.1186/s13256-022-03399-3 35641986 PMC9153143

[mgg32249-bib-0023] Shao, H. , Hua, J. , Wu, Q. , Li, X. , Zhang, M. , Wang, H. , Wu, J. , Xu, L. , Xie, Y. , Li, L. , & Chen, H. (2020). Identification of a mutation in the novel compound heterozygous CFTR in a Chinese family with cystic fibrosis. Canadian Respiratory Journal, 2020, 6507583. 10.1155/2020/6507583 32454915 PMC7229557

[mgg32249-bib-0024] Smirnikhina, S. A. , Kondrateva, E. V. , Adilgereeva, E. P. , Anuchina, A. A. , Zaynitdinova, M. I. , Slesarenko, Y. S. , Ershova, A. S. , Ustinov, K. D. , Yasinovsky, M. I. , Amelina, E. L. , Voronina, E. S. , Yakushina, V. D. , Tabakov, V. Y. , & Lavrov, A. V. (2020). P.F508del editing in cells from cystic fibrosis patients. PLoS ONE, 15(11), e0242094. 10.1371/journal.pone.0242094 33175893 PMC7657551

[mgg32249-bib-0025] Tabaripour, R. , Niaki, H. A. , Douki, M. R. , Bazzaz, J. T. , Larijani, B. , & Yaghmaei, P. (2012). Poly thymidine polymorphism and cystic fibrosis in a non‐Caucasian population. Disease Markers, 32(4), 241–246. 10.3233/DMA-2011-0880 22430190 PMC3826484

[mgg32249-bib-0026] Wang, H. , An, M. , Liu, Y. , Hu, K. , Jin, Y. , Xu, S. , Chen, B. , & Lu, M. (2020). Genetic diagnosis and sperm retrieval outcomes for Chinese patients with congenital bilateral absence of vas deferens. Andrology, 8(5), 1064–1069. 10.1111/andr.12769 32020786

[mgg32249-bib-0027] Wu, C. C. , Hsieh‐Li, H. M. , Lin, Y. M. , & Chiang, H. S. (2004). Cystic fibrosis transmembrane conductance regulator gene screening and clinical correlation in Taiwanese males with congenital bilateral absence of the vas deferens. Human Reproduction, 19(2), 250–253. 10.1093/humrep/deh073 14747162

[mgg32249-bib-0028] Yu, J. , Chen, Z. , Ni, Y. , & Li, Z. (2012). CFTR mutations in men with congenital bilateral absence of the vas deferens (CBAVD): A systemic review and meta‐analysis. Human Reproduction, 27(1), 25–35. 10.1093/humrep/der377 22081250

[mgg32249-bib-0029] Zhao, X. , Liu, K. , Xu, W. , Xiao, M. , Zhang, Q. , Song, J. , Chen, K. , Liu, Y. , Tian, X. , Xu, K. F. , & Zhang, X. (2022). Novel mutation c.1210‐3C > G in cis with a poly‐T tract of 5T affects CFTR mRNA splicing in a Chinese patient with cystic fibrosis. Frontiers in Medicine, 16(1), 150–155. 10.1007/s11684-021-0846-5 34302615

