# Supplementary figures and images for: Compound heterozygous variants in CFTR with potentially reducing ATP‐binding ability identified in Chinese infertile brothers with isolated congenital bilateral absence of vas deferens
Source: Mol Genet Genomic Med. 2023 Jul 24;11(11):e2249. doi: 10.1002/mgg3.2249 (PMC10655520; doi:10.1002/mgg3.2249)

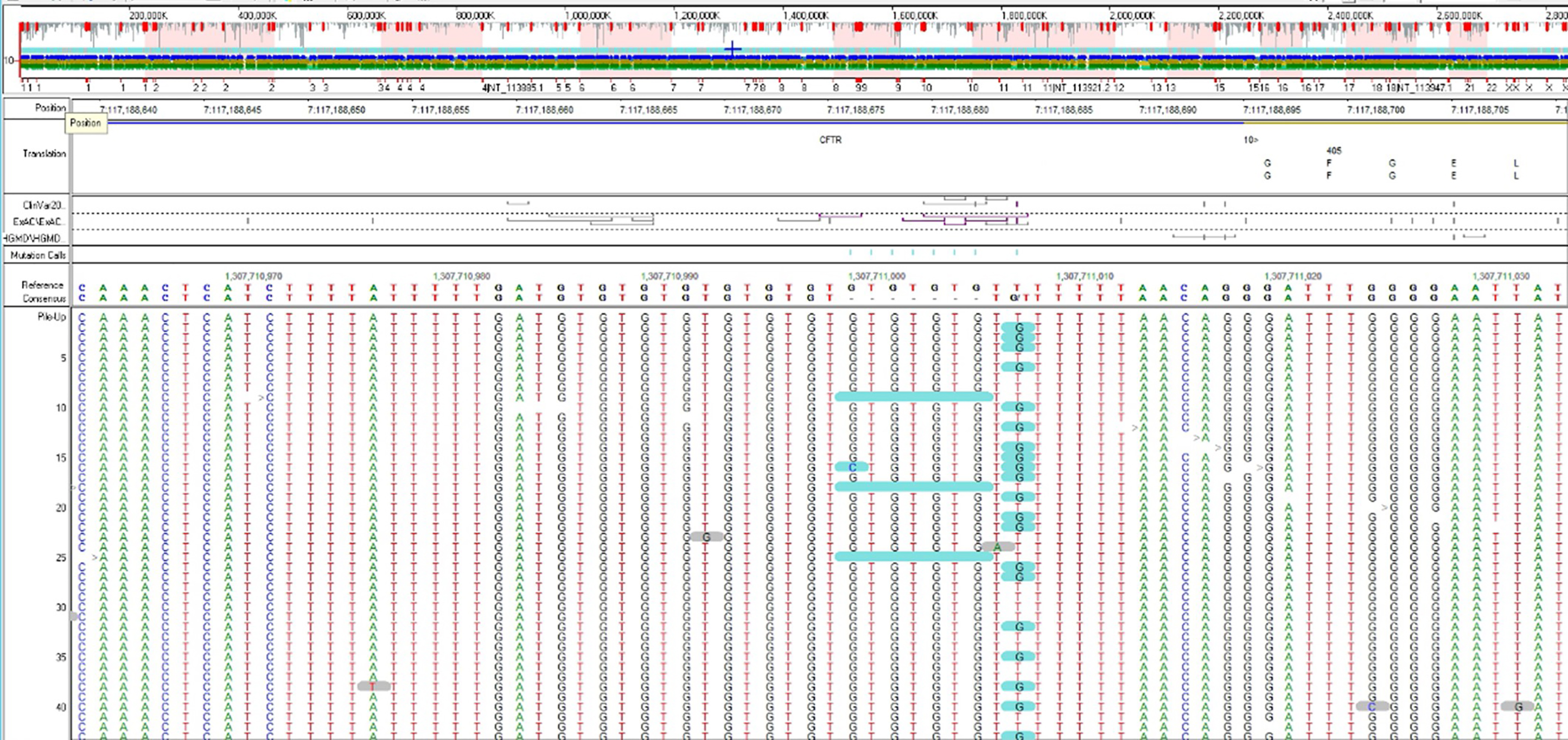

Supplement: Supplementary file 1 — Figure S1 [file MGG3-11-e2249-s004.jpg]

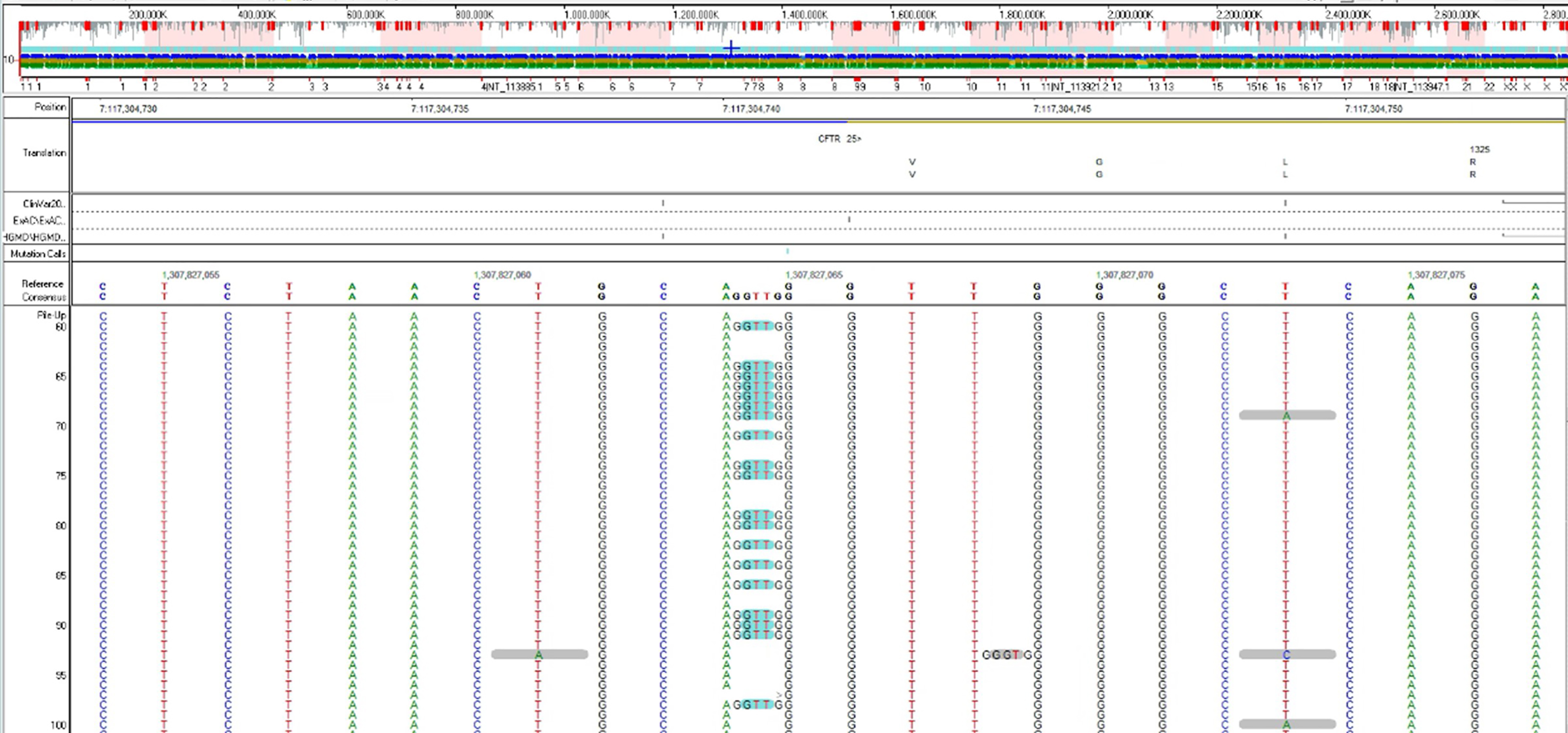

Supplement: Supplementary file 2 — Figure S2 [file MGG3-11-e2249-s003.jpg]

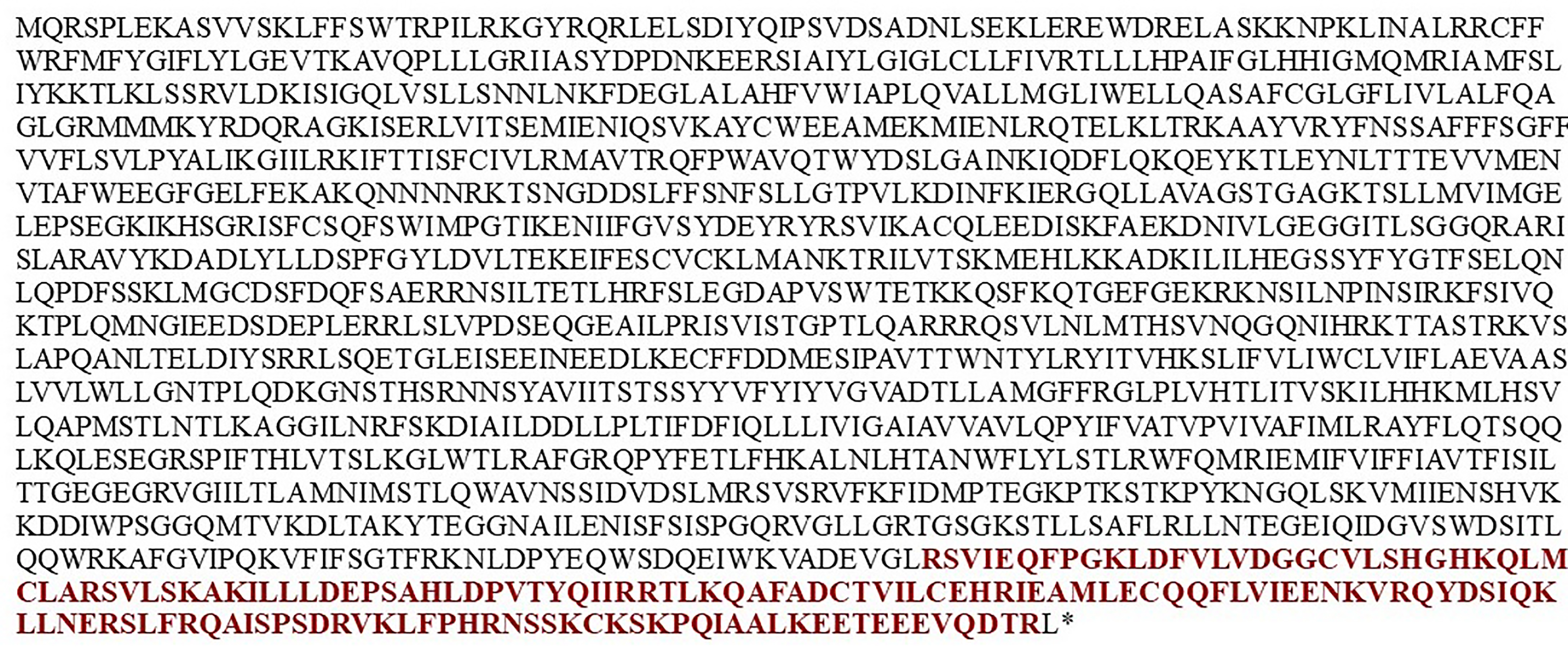

Supplement: Supplementary file 3 — Figure S3 [file MGG3-11-e2249-s002.jpg]

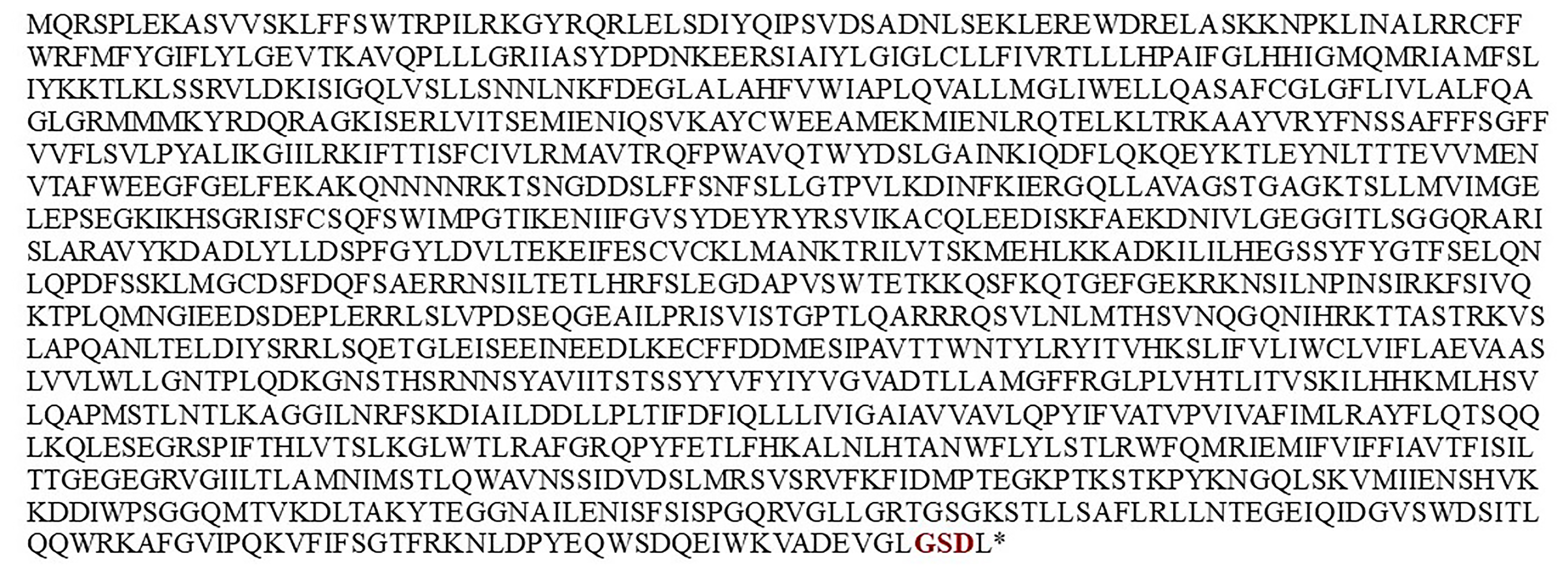

Supplement: Supplementary file 4 — Figure S4 [file MGG3-11-e2249-s001.jpg]
